# Increased left anterior insular and inferior prefrontal activity in post-stroke mania

**DOI:** 10.1186/1471-2377-12-68

**Published:** 2012-08-06

**Authors:** Akihiro Koreki, Keisuke Takahata, Hajime Tabuchi, Motoichiro Kato

**Affiliations:** 1 Department of Neuropsychiatry, Ashikaga Red Cross Hospital, Tochigi, Japan; 2 Department of Neuropsychiatry, Keio University School of Medicine, 35 Shinanomachi, Shinjuku-ku, Tokyo, 160-8582, Japan; 3 Molecular Neuroimaging Program, Molecular Imaging Center, National Institute of Radiological Sciences, Chiba, Japan

**Keywords:** Contralesional release phenomenon, Insula, Mania, SPECT, Stroke

## Abstract

**Background:**

Post-stroke mania is an infrequent complication after stroke, and the mechanisms underlying this disorder remain unclear. Although a contralesional release phenomenon has been implicated in post-stroke mania, empirical findings are lacking.

**Case presentation:**

We present a case report of post stroke mania. Single photon emission tomography (SPECT) was performed twice, during the manic state and during the remitted euthymic state. The first SPECT study performed during the manic state demonstrated hypoperfusion in the right temporal and frontal regions due to right putaminal hemorrhage. It also showed hyperperfusion in the inferior lateral prefrontal lobe, the temporal lobe, and the medial and lateral parts of the parietal lobe in the left hemisphere. The second SPECT study performed during the euthymic state demonstrated moderate improvement in the hypoperfusion in the right fronto-temporal regions. Furthermore, compared to the findings on the first SPECT study, the second study showed that the focal hyperperfusion in the anterior insular cortex, inferior lateral prefrontal lobes, and superior-middle temporal gyrus in the left hemisphere had vanished.

**Conclusion:**

Increased left inferior prefrontal and anterior insular activity and reduced extensive right fronto-temporal lobe activity are involved in the development of post-stroke mania.

## Background

Depression is a common neuropsychiatric consequence of stroke. In contrast, post-stroke mania is less common after stroke, and the mechanisms of this disorder remain unclear [1]. Findings from previous neuroimaging studies demonstrated that post-stroke mania is more often associated with lesions in the right hemisphere than in the left hemisphere [2-4]. One single photon emission tomography (SPECT) study showed that a patient with post-stroke mania had a unique pattern of left orbito-frontal hyperperfusion with extensive right frontal hypoperfusion [5]. This finding indicated that manic symptoms after stroke could be caused by a contralesional release phenomenon. In other words, the interruption of inter-hemispheric inhibition could give rise to increased activation of the left hemisphere after right hemisphere damage. However, few neuroimaging studies have directly investigated the relationship between changes in regional cerebral blood flow and the clinical course of post-stroke mania. In the present study, to investigate mechanisms of post-stroke mania, changes in regional cerebral blood flow were examined using SPECT studies during both the manic state and the remitted euthymic state on separate days in a patient with post-stroke mania following a right putaminal hemorrhage. We hypothesized that the regional cerebral blood flow in the manic state demonstrates right fronto-temporal lobe hypoperfusion due to the brain hemorrhage itself and hyperperfusion in the left orbito-fronto-temporo-parietal regions resulting from contralesional release phenomenon, and that this increased activity of the left hemisphere would resolve into the normal state after recovery from post-stroke mania.

## Case presentation

The patient was a 68-year-old, right-handed male office worker who had retired at the age of 62 years. He had a past history of hypertension, hyperlipidemia, and gout. He had no prior psychiatric history, and no family history of psychiatric disorders. His medication at the time was digoxin, atenolol, candesartan, and atorvastatin.

**Figure 1:**
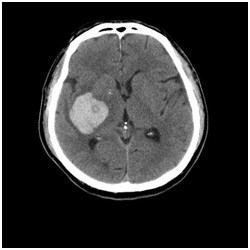
Right putaminal hemorrhage in a patient with post-stroke mania.

His premorbid personality before the stroke was taciturn, serious, and not cyclothymic. In January 2009, he suffered a right putaminal hemorrhage [Figure 1], which led to left hemi paralysis. He was admitted to our hospital for treatment and then transferred to another hospital for rehabilitation. After rehabilitation, his left hemi paralysis improved without any motor complications. Six weeks after the stroke, however, he developed severe manic symptoms and was referred to our clinic.

He presented with elevated mood, irritability, motor excitement, and pressure of speech with racing thoughts. He was euphoric and always smiling. Since he talked too fast and his conversational content fluctuated, it was difficult to understand what he said. He got angry over trivial events and often threw a newspaper at his wife. He also had psychomotor agitation and frequently tried to see estranged friends without a specific reason. He could not stop talking even when ordered to do so. Moreover, he also displayed less need for sleep. He believed that he could get along just fine on a few hours of sleep at night. These symptoms met the clinical criteria for a manic episode according to the DSM-IV-TR criteria [6]. His Young Mania Rating Scale (YMRS) [7] was 42 points, which indicated a severe manic state.

His consciousness level was clear. The findings on his electroencephalogram were normal. Clinical neuropsychological tests were performed during the manic state. The patient’s full-scale intelligence quotient (FIQ) on the Wechsler Adult Intelligence Scale III was 102 (verbal IQ 113, performance IQ 88). The Wechsler Memory Scale-Revised (WMS-R) revealed that he had mild memory deficits (general memory index 91, visual memory index 97, verbal memory index 90, delayed memory index 75). On executive function testing, his performances were normal on the Wisconsin Card Sorting Test, Modified Stroop Test, and the Word Fluency Test. Taken together, the neuropsychological assessments demonstrated a mild memory disorder with a slightly low PIQ and intact executive function.

The patient was treated at our hospital as an outpatient. He was started on sodium valproate, and the dose was gradually increased to 600 mg/day. Olanzapine 7.5 mg/day was also introduced. After 2 months, he showed considerable improvement, and the YMRS score dropped from 42 to 8. His manic symptoms diminished 2 years after stroke onset, and the YMRS score decreased to 0 points. During the course of post-stroke emotional impairment, he did not have a depressive state.

Single photon emission tomography (SPECT) was performed twice on separate days: during the manic state (YMRS 42 points) before treatment, and during the remitted state (YMRS 0 points) after treatment, with an interval of 2 years. At the time of first SPECT scan, he took sodium valproate 600 mg/day and olanzapine 5 mg/day, and at the time of second SPECT scan, he continued to take sodium valproate 600 mg/day and did not take any antipsychotic drug.

Projection data were acquired 30 minutes after an i.v. bolus injection of 740 MBq of 99mTc-ECD using a 3-headed rotating gamma camera (Toshiba GCA-9300A/DI; Toshiba Corporation, Tokyo, Japan) equipped with an ultra high-resolution fan beam collimator, and a medical image processor (GMS5500U/DI; Toshiba Corporation, Tokyo, Japan) was used image processing. The energy window for acquisition was set at 140 keV, with a width of 20%. The gamma camera was rotated continuously for 16 minutes, and SPECT data were arranged into 90 projection angles over 360 degrees. Images were reconstructed in a 128 x 128 matrix using a ramp filter after the data were processed with a Butterworth filter (order 8, 0.12 cycles/pixel). SPECT images were spatially normalized to standardized stereotactic space, smoothed using a 12-mm FWHM isotropic Gaussian kernel, and analyzed using the Easy Z-Score Imaging System (eZIS) [8]. Each SPECT image was compared with the mean and SD of SPECT images of 20 age-matched healthy male controls using voxel-by-voxel Z-score analysis after normalization to global mean voxel values; Z-score = ([control mean]_ [individual value])/(control SD). The Z-score maps were displayed by projection with an averaged Z-score of 14-m thickness to a surface rendering of the anatomically standardized MRI template with a two-tailed view of a hot color scale (hyper perfusion, Z-score level >2.0) and a cold color scale (hypo perfusion, Z-score level >2.0). The extent threshold of the cluster size was set at z300 voxels.

**Figure 2:**
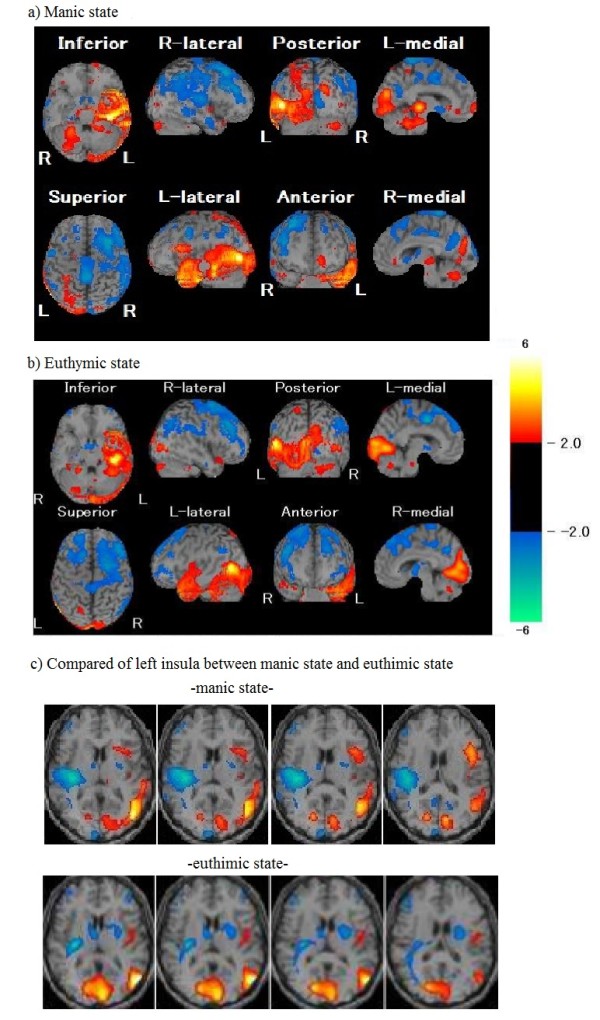
SPECT images obtained during a) the manic state and b) the euthymic state, and c) Comparison of regional cerebral blood flow of the left anterior insula between the manic state and the euthymic state.

The first SPECT study performed during the manic state demonstrated that the patient had hypoperfusion in the right temporal and frontal regions due to right putaminal hemorrhage [Figure 2]. It also revealed hyperperfusion in the inferior lateral prefrontal lobes, the temporal lobe, and the medial and lateral parts of the parietal lobes in the left hemisphere. The second SPECT study performed during the euthymic state demonstrated moderate improvement of the hypoperfusion in the right fronto-temporal regions. Furthermore, compared to the findings on the first SPECT study, the second study showed that the focal hyperperfusion had vanished in the anterior insular cortex, inferior lateral prefrontal lobes, and superior-middle temporal gyrus in the left hemisphere.

Informed consent was obtained from this patient.

## Discussion and conclusions

To the best of our knowledge, this is the first case report to demonstrate the relationship between the contralesional release phenomenon and the clinical symptoms of post-stroke mania with direct comparison of regional cerebral blood flow during the manic state with that during the remitted euthymic state.

Before we discuss the possible implication of our findings, several diagnostic issues should be considered. The present patient clearly suffered from post-stroke mania, and his symptoms met the clinical criteria for a manic episode according to DSM-IV-TR criteria [6]. His symptoms were not a disinhibition syndrome after stroke, because he had a distinct elevated mood without a dysphoric state, and neuropsychological examination revealed that he had normal executive function. Normal findings on his electroencephalogram provided evidence that epilepsy was not involved in his manic symptoms. Furthermore, the development of the manic state could not be explained by his positive psychological state due to recovery of neurological symptoms or as a manic defence to the experience of stroke, because his distinct elevated mood and other manic symptoms were too long continued and at a pathological level. Therefore, we concluded that he suffered from post-stroke mania with brain dysfunction.

The first SPECT study demonstrated a unique pattern of left fronto-temporo-parietal hyperperfusion with extensive right fronto-temporal hypoperfusion during manic symptoms. This pattern was consistent with that due to a contralesional release phenomenon after brain lesions, and it was similar to a past case report [5]. Interestingly, this imbalance in the regional cerebral blood flow improved after recovery of his manic symptoms. In particular, the increased brain activity in the inferior lateral prefrontal lobes, anterior insular cortex, and superior-middle temporal gyrus in the left hemisphere disappeared in the remitted euthymic state.

Results of SPECT studies in the present case were consistent with previous findings from functional neuroimaging studies of primary mania. For example, Blumberg et al. demonstrated decreased right prefrontal cortex activation and increased left prefrontal cortex activation in patients with primary mania using PET [9,10]. They proposed that primary mania is associated with an imbalance in prefrontal activity, with right less than left, and ventral less than dorsal [10]. Similarly, other researchers showed a pattern of greater left than right regional cerebral blood flow in the inferior prefrontal cortex in acute mania [11], and they suggested that manic symptoms are related to relatively increased left frontal activity [12]. It has also been reported that greater left than right frontal cortical activity is associated with positive affect and approach motivation, which can generate a manic state [13]. Thus, it is plausible that the imbalance in brain perfusion, reduced right fronto-temporal activity, and increased left inferior prefrontal activity may have contributed to the development of manic symptoms in the present patient.

In the present case, the hyperperfusion in the left anterior insular cortex vanished after recovery from mania. Hyperperfusion in the left anterior insular cortex was shown in a previous case report [5]. The anterior insular cortex may be implicated in emotional awareness [14]. Several neuroimaging studies of emotional awareness reported joint activation of the anterior insular cortex and the anterior cingulate cortex in the subjective experience of emotional feelings, including happiness, sexual arousal, and empathy. Furthermore, activation of the left anterior insular cortex was reported in subjects hearing pleasant music, seeing a smile, and experiencing joy [14]. Clinical neurophysiologic studies demonstrated that intensive positive feelings in ecstatic seizures can originate from the left anterior insula, a region that has been suggested to engender self-awareness associated with positive feelings [15,16]. In addition, it has been proposed that the right anterior insular cortex is closely associated with negative emotion, while the left anterior insular cortex is associated with positive emotion [14]. Therefore, in the present patient, the hyperactivation in the left anterior insular cortex may have led to the patient’s pathologically elevated mood, which is the core symptom of mania. Moreover, the hypoactivation in the right anterior insular cortex in this patient might have prevented him from having negative emotions, which exacerbated his manic symptoms.

In the present study, we observed the treatment effect of sodium valproate and olanzapine in improving the manic symptoms. Therefore, effect of drugs on the regional cerebral blood flow should be considered. This patient took olanzapine at the time of first SPECT scan, and sodium valproate at the time of both SPECT scans. It was reported that olanzapine does not affect regional cerebral blood flow [17]. Furthermore, it was also shown that sodium valproate diminishes global cerebral blood flow in wide areas of the brain [18]. Based on these findings, it is difficult to determine whether there is any kind of treatment effect or not. It is still unclear whether these regional changes are related to the cessation of olanzapine or to a possible effect of sodium valproate. In order to evaluate this, there is need for another study design including the pre and post treatment effect of both drugs (olanzapine and sodium valproate), although there are restricted studies in this topic.”

In conclusion, the present case showed that increased left inferior prefrontal and anterior insular activity and reduced right fronto-temporal lobe activity was involved in the development of post-stroke mania, possibly due to an interhemispheric prefrontal and insular imbalance with a contralesional release phenomenon.

## Competing interests

The authors declare no competing interests.

## Authors’ contributions

MK and HT cared for the patient, and participated in the design of the case report and neuroimaging research. AK, KT, HT and MK analyzed SPECT data and wrote the paper. All authors read and approved the final manuscript.

## Pre-publication history

The pre-publication history for this paper can be accessed here:

http://www.biomedcentral.com/1471-2377/12/68/prepub

## References

[B1] Ferro JM Caeiro L Santos C Poststroke emotional and behavior impairment: A narrative review Cerebrovasc Dis 2009 27 suppl 1 197 203 19342852 10.1159/000200460

[B2] Braun CM Larocque C Daigneault S Montour-Proulx I Mania, pseudomania, depression, and pseudodepression resulting from focal unilateral cortical lesions, Neuropsychiatry Neuropsychol Behav Neurol 1999 12 35 51 10082332

[B3] Carran MA Kohler CG O'Connor MJ Bilker WB Sperling MR Mania following temporal lobectomy Neurology 2003 61 770 774 10.1212/01.WNL.0000086378.74539.85 14504319

[B4] Robinson RG Boston JD Starkstein SE Price TR Comparison of mania and depression after brain injury: causal factors Am J Psychiatry 1988 145 172 178 3341462 10.1176/ajp.145.2.172

[B5] Mimura M Nakagome K Hirashima N Ishiwata H Kamijima K Shinozuka A Matsuda H Left frontotemporal hyperperfusion in a patient with post-stroke mania Psychiatry Res 2005 139 263 267 10.1016/j.pscychresns.2005.05.007 16054342

[B6] DSM-IV Diagnostic and Statistical Manual of Mental Disorders 2000 4 Washington D.C: American Psychiatric Association text revision edition

[B7] Young RC Biggs JT Ziegler VE Meyer DA A rating scale for mania: reliability, validity and sensitivity Br J Psychiatry 1978 133 429 435 10.1192/bjp.133.5.429 728692

[B8] Matsuda H Mizumura S Souma T Takemura N Conversion of brain SPECT images between different collimators and reconstruction processes for analysis using statistical parametric mapping Nuclear Medical Communication 2004 25 67 74 10.1097/00006231-200401000-00010 15061267

[B9] Blumberg HP Stern E Ricketts S Martinez D de Asis J White T Epstein J Isenberg N McBride PA Kemperman I Emmerich S Dhawan V Eidelberg D Kocsis JH Silbersweig DA Rostral and orbital prefrontal cortex dysfunction in the manic state of bipolar disorder Am J Psychiatry 1999 156 1986 1988 10588416 10.1176/ajp.156.12.1986

[B10] Blumberg HP Stern E Martinez D Ricketts S de Asis J White T Epstein J McBride PA Eidelberg D Kocsis JH Silbersweig DA Increased anterior cingulate and caudate activity in bipolar mania Biol Psychiatry 2000 48 1045 1052 10.1016/S0006-3223(00)00962-8 11094137

[B11] Rubin E Sackeim HA Prohovnik I Moeller JR Schnur DB Mukherjee S Regional cerebral blood flow in mood disorders: IV comparison of mania and depression Psychiatry Res 1995 61 1 10 10.1016/0925-4927(95)02594-N 7568564

[B12] Harmon-Jones E Abramson LY Sigelman J Bohlig A Hogan ME Harmon-Jones C Proneness to hypomania/mania symptoms or depression symptoms and asymmetrical frontal cortical responses to an anger-evoking event J Per Soc Psychol 2002 82 610 618 11999927

[B13] Harmon-Jones E Gable PA Peterson CK The role of asymmetric frontal cortical activity in emotion-related phenomena: a review and update Biol Psychol 2010 84 451 462 10.1016/j.biopsycho.2009.08.010 19733618

[B14] Craig AD How do you feel — now? The anterior insula and human awareness Nat Rev Neuroscience 2009 10 59 70 10.1038/nrn2555 19096369

[B15] Landtblom AM Lindehammar H Karlsson H Craig AD Insular cortex activation in a patient with "sensed presence"/ecstatic seizures Epilepsy Behav 2011 20 714 718 10.1016/j.yebeh.2011.01.031 21440512

[B16] Picard F Craig AD Ecstatic epileptic seizures: A potential window on the neural basis for human self-awareness Epilepsy Behav 2009 16 539 546 10.1016/j.yebeh.2009.09.013 19836310

[B17] Gonul AS Kula M Sofuoglu S Tutus A Esel E Tc-99 HMPAO SPECT study of regional cerebral blood flow in olanzapine-treated schizophrenic patients Eur Arch Psychiatry Clin Neurosci 2003 253 29 33 10.1007/s00406-003-0401-1 12664310

[B18] Gaillard WD Zeffiro T Fazilat S DeCarli C Theodore WH Effect of valproate on cerebral metabolism and blood flow: an 18 F-2-deoxyglucose and 15O water positron emission tomography study Epilepsia 1996 37 515 521 10.1111/j.1528-1157.1996.tb00602.x 8641226

